# Closed solutions to a differential-difference equation and an associated plate solidification problem

**DOI:** 10.1186/s40064-016-2915-9

**Published:** 2016-08-05

**Authors:** Olawanle P. Layeni, Adegbola P. Akinola, Jesse V. Johnson

**Affiliations:** 1 Department of Mathematics, Obafemi Awolowo University, Ile-Ife, 220005 Nigeria; 2 Department of Computer Science, University of Montana, Missoula, MT 59812 USA

**Keywords:** Differential-difference equation, Clarkson–Kruskal’s method, Stefan problem, Primary 80A22; Secondary 34A33

## Abstract

Two distinct and novel formalisms for deriving exact closed solutions of a class of variable-coefficient differential-difference equations arising from a plate solidification problem are introduced. Thereupon, exact closed traveling wave and similarity solutions to the plate solidification problem are obtained for some special cases of time-varying plate surface temperature.

## Background

In a recent paper by Grzymkowski et al. (2013), an analytical method for finding the approximate temperature distribution in a solidifying plate modeled by the one-phase problem

1a $$\begin{aligned} \partial _{t}T&=b \partial _{xx}T \qquad {\mathrm{in}}\,\left( \Phi (t),{\bar{x}}\right) \times \left( 0,\tau \right) ; \end{aligned}$$

1b $$\begin{aligned} \Phi (t)&={\bar{x}}-\xi (t), \qquad \xi (0)=0 ; \end{aligned}$$

1c $$\begin{aligned} T\Big |_{x={\bar{x}}}&=\Psi (t) \qquad {\mathrm{for}}\,t\in \left[ 0,\tau \right] ; \end{aligned}$$

1d $$\begin{aligned} T\Big |_{x=\Phi (t)}&=T^{\star }\in {\mathbb {R}}\, \qquad {\mathrm{for}}\,t\in \left[ 0,\tau \right] ; \end{aligned}$$

1e $$\begin{aligned} -\lambda \partial _{x}T\Big |_{x=\Phi (t)}&=\gamma \kappa d_{t}\Phi (t) \qquad {\mathrm{for}}\,t\in \left[ 0,\tau \right] , \end{aligned}$$

was introduced. Equation (1), which models the temperature distribution only in the solid phase of the plate is such that ${\bar{x}}$ is the half of the plate thickness, $\lambda$ its thermal conductivity, $\kappa$ its latent heat of fusion, $\gamma$ its mass density and *b* its diffusivity given by $b=\lambda /(\gamma \kappa )$. Further, $\Phi (t)$ describes the time-dependent position of the solidification front, $\xi (t)$ the time-dependent thickness of the solidified layer, $\Psi (t)$ is the time-dependent temperature of the plate’s surface, and $\tau$ the duration for complete solidification. The approach taken by Grzymkowski and coauthors relied on the assumption that the unknown temperature field $T=T(x,t)$ takes the form of an exponential generating function

2 $$\begin{aligned} T(x,t)=\sum _{j=0}^{\infty } A_{j}(t)\frac{\left( x-\Phi (t)\right) ^{j}}{j!} , \end{aligned}$$

with the undetermined sequence ${\left\{ A_{j}(t)\right\} _{0}^{\infty }}$ and solidification front $\Phi (t)$ [through $\xi (t)$] satisfying the boundary conditions

3a $$\begin{aligned} A_{0}(t)&=T^{\star }; \end{aligned}$$

3b $$\begin{aligned} A_{1}(t)&=\frac{\gamma \kappa }{\lambda }\xi ^{'}(t) \qquad {\mathrm{for}}\,t\in \left[ 0,\tau \right] ; \end{aligned}$$

3c $$\begin{aligned} \Psi (t)&=\sum _{j=0}^{\infty } A_{j}(t)\frac{\xi ^{j}(t)}{j!} \qquad {\mathrm{for}}\,t\in \left[ 0,\tau \right] , \end{aligned}$$

where in Eq. (3b) and through the rest of this paper, the prime is indicative of an ordinary derivative with respect to the temporal variable *t*.

This technique, which is plausible provided series (2) is convergent in the interval $\left( \Phi (t),{\overline{x}}\right)$ for all $t\in \left( 0,\tau \right)$, leads to the reformulation of Problem (1) as that of finding such $A_{j}(t)$ and $\xi (t)$ as would satisfy the variable coefficient differential-difference equation $({\mathcal {D}}\Delta {\mathcal {E}})$

4 $$\begin{aligned} A_{j}^{'}(t)+\xi ^{'}(t)A_{j+1}(t)-bA_{j+2}(t)=0 \quad {\mathrm{in}}\,{{\mathbb {Z}}}^{+}\cup \left\{ 0\right\} \times (0,\tau ) . \end{aligned}$$

${{\mathbb {Z}}}^{+}$ is the set of positive integers.

${\mathcal {D}}\Delta {\mathcal {E}}$s of similar structures to Eq. (4) with both constant and variable coefficients have been studied in the literature by various methods. Numerical and truncation techniques were deployed by were deployed by Barry (1966) in studying a first order constant coefficient first order ${\mathcal {D}}\Delta {\mathcal {E}}$. Feynman-Dyson (Feynman 1951) and Magnus (Blanes et al. 2009) time-ordering techniques have been employed in solving constant and variable coefficient Raman–Nath equations (which constitute a class of first order ${\mathcal {D}}\Delta {\mathcal {E}}$s) in Bosco and Dattoli (1983), Bosco et al. (1984), Dattoli et al. (1984), Dattoli et al. (1985). Alimohomadi et al. (2012) solved a variable coefficient ${\mathcal {D}}\Delta {\mathcal {E}}$ using the Wei-Norman Lie-algebraic time ordering method of Wei and Norman (1963), Shang (2012).

The discrete version of Lie-group symmetric reduction was introduced by Levi and Winternitz (1991), Levi and Winternitz (1993). This novelty afforded the study of symmetry reductions of several classes of differential-difference equations: Shen (2007) used a combination of the classical Lie-group method and symbolic computation to solve nonlinear constant coefficient ${\mathcal {D}}\Delta {\mathcal {E}}$s; Shen et al. (2004) derived symmetry reductions of Toda-like lattice equations by a similar method; while Li et al. (2008) and Lv et al. (2011) deployed a synthesis of the Lie group and Harrison-Estabrook geometric techniques in the study of Lie symmetries of some differential-difference equations. The technique employed in this paper derives partly from that of Shen (2005) in which the direct similarity method of Clarkson and Kruskal (1989) was extended to the study of ${\mathcal {D}}\Delta {\mathcal {E}}$s.

Solidification of materials has been extensively studied in the literature, see Kurz and Fisher (1992), Dantzig and Rappaz (2009), Glicksman (2011) and the references therein, and still receives continuous attention due to its huge significance to industrial processes. Several mathematical techniques have been developed and exploited in the study of models similar to  (1); Chuang and Szekely (1971) proposed a Green’s function-based semi-analytical method for studying approximate solutions of a solidification problem; Charach and Zoglin (1985) employed a combination of the heat balance integral method (Goodman 1958; Mitchell and Myers 2010; Layeni and Johnson 2012) and time-dependent perturbation theory to construct approximate solutions for solidification of a finite slab which is valid for small Stefan numbers and uniformly in time; Prud’homme et al. (1989) investigated heat transfer during the solidification of materials in various geometries by the method of strained coordinates; and Gonzalez et al. (2003) developed a computational simulation system for modelling a solidification process during continuous casting.

Grzymkowski et al. resorted to deriving approximate analytical and numerical solutions for Eq. (4), and consequently Eq. (1), primarily due to the difficulty encountered in obtaining closed-form solutions satisfying boundary conditions Eq. (3). Consequent upon the inability of their approach at solving ${\mathcal {D}}\Delta {\mathcal {E}}$ (4) exactly and partially for the sake of completeness we revisit the problem, proffering two efficient protocols for solving (4).

The objective of this paper is twofold. The one is to construct exact solutions to ${\mathcal {D}}\Delta {\mathcal {E}}$ (4) through two distinct syntheses of the ideas of Shen (2005), Clarkson and Kruskal (1989) or Bateman (1943). The other is to apply the obtained results in establishing exact closed-form solutions to the plate solidification problem (1).

The rest of this paper is organized as follows: The second Section gives the similarity reductions and closed form solutions to ${\mathcal {D}}\Delta {\mathcal {E}}$ (4). The third Section gives two distinct classes of solutions to the solidification process courtesy the results of the second, while the last concludes the paper.

## Clarkson–Kruskal’s similarity reduction and explicit solutions

In this Section, in line with the direct method of Clarkson and Kruskal (1989), we seek solutions to ${\mathcal {D}}\Delta {\mathcal {E}}$ (4) which are of the form

5 $$\begin{aligned} A_{j}(t)=\Upsilon (t)+\Theta (t)V_{j}(z) , \quad z=z(t) , \end{aligned}$$

with $\Upsilon$, $\Theta$, $V_{j}$ and *z* being continuously differentiable functions.

Substituting Eq. (5) into Eq. (4) yields

6 $$\begin{aligned} \Theta (t)z^{'}(t)\frac{dV_{j}(z)}{dz}&+\Theta ^{'}(t)V_{j}(z)+\Theta (t)\xi ^{ '}(t)V_{j+1}(z)\nonumber \\&-b\Theta (t)V_{j+2}(z)+\left( \Upsilon ^{'}(t)+ \left[ \xi ^{ '}(t)-b\right] \Upsilon (t)\right) =0 . \end{aligned}$$

### Clarkson–Kruskal similarity reduction

As a result of the variant of Clarkson–Kruskal’s direct method due to Shen (2005), the following system of equations can be sieved from Eq. (6):

7a $$\begin{aligned} \Theta ^{'}(t)&=\Theta (t)z^{'}(t)\Gamma _{1}(z(t)) ; \end{aligned}$$

7b $$\begin{aligned} \xi ^{ '}(t)&=z^{'}(t)\Gamma _{2}(z(t)) ; \end{aligned}$$

7c $$\begin{aligned} -b&=z^{'}(t)\Gamma _{3}(z(t)); \end{aligned}$$

7d $$\begin{aligned} \Theta (t)z^{'}(t)\Gamma _{4}(z(t))&=\Upsilon ^{ '}(t)+\xi ^{ '}(t)\Upsilon (t)-b\Phi (t) , \end{aligned}$$

$\Gamma _{j}$, $j\in \{1,2,3,4\}$ being undetermined functions of *z*(*t*) defined such that (Shen 2005),

(i) if a function *R*(*t*) is found to have the form $R(t)=Q(t)\Gamma _{j}(z(t))$ then one may set $\Gamma _{j}(z(t))=1$ ;
(ii) if a function *S*(*t*) is found to have the form $S(t)=T(t)+U(t)\Gamma _{j}(z(t))$ then one may set $\Gamma _{j}(z(t))=0$ ; and
(iii) if *z*(*t*) is determined from the implicit equation $f(z(t))=g(t)$, *f* invertible then one may set $f(z)=z(t)$,

where *Q*, *T*, *U* and *g* are known functions with sufficient regularity.

Proceeding from Eq. (7a), it is observed that $$\Theta ^{'}(t)\Theta ^{-1}(t)=$$$$z^{ '}(t)\Gamma _{1}(z(t))=$$$$z^{'}(t)\Gamma _{1}^{'}(z(t))=$$$$\left( \Gamma _{1}(z(t))\right) ^{'}$$, $$\Gamma _{1}(z(t))$$ being equivalent to $$\Gamma _{1}^{'}(z(t))$$, see Shen (2005). This implies that $$\left( \log (\Theta (t))\right) ^{'}=\left( \Gamma _{1}(z(t))\right) ^{'}$$, which further means that $$\Theta (t)=\Theta _{0}\exp \left( \Gamma _{1}(z(t))\right) \Leftrightarrow$$$$\Theta (t)=\Theta _{0}= constant$$ and $$\Gamma _{1}(z(t))=0$$. Applying these results to Eqs. (7b) and  (7c) shows that $$\xi ^{ '}(t)=z^{ '}(t)\Gamma _{2}(z(t))$$ implies that $$\xi ^{'}(t)=z^{ '}(t)$$ and $$\Gamma _{2}(z(t))=1$$.

Also, $-b=z^{'}(t)\Gamma _{3}(z(t))=\xi ^{'}(t)\Gamma _{3}(z(t))$ or $-b=-z^{'}(t)\Gamma _{10}(z(t))=-\xi ^{'}(t)\Gamma _{10}(z(t))$, $\Gamma _{10}(z(t))=-\Gamma _{3}(z(t))$. These imply that $\xi ^{ '}(t)=\pm b$ and $\Gamma _{3}(z(t))=1$. Since $\xi ^{'}(t)=\pm b$, then $\xi (t)=\pm bt+\gamma$, $\gamma \in {{\mathbb {R}}}$. Further, $z^{'}(t)=\xi ^{'}(t)$ implies that $z(t)=\xi (t)+\gamma ^{*}=\pm bt+\varpi$, where $\varpi =\gamma +\gamma ^{*}\in {{\mathbb {R}}}$. Setting $\Gamma _{4}(z(t))=0$ in (7d) implies that $\Upsilon ^{ '}(t)\Upsilon ^{-1}(t)=b-\xi ^{ '}(t)=$ 0 or 2*b*, showing that $\Upsilon (t)$ is either a real constant $\Upsilon _{0}$ or equal to $\Upsilon _{0}\exp (2bt)$.

As a consequence of the above, ${\mathcal {D}}\Delta {\mathcal {E}}$  (4) has solutions

8 $$A_{j}(t)=\Theta _{0}V_{j}(z(t))+ {\left\{ \begin{array}{ll} \Upsilon _{0}&\quad z(t)=\xi (t)=bt+\varpi ,\\ \Upsilon _{0}\exp (2bt)& \quad z(t)=\xi (t)=-bt+\varpi ; \end{array}\right. }$$

$\Upsilon _{0},\Theta _{0},b,\varpi$ real constants; $V_{j}(z(t))$ being solutions of the constant coefficient differential-difference equations

9 $$\begin{aligned} {\left\{ \begin{array}{ll} {\displaystyle \frac{dV_{j}(z(t))}{dz}+V_{j+1}(z(t))\pm V_{j+2}(z(t))=0 ;}\\ \xi (t)=\mp bt+\varpi .\\ \end{array}\right. } \end{aligned}$$

Equations (8) and (9) constitute a Clarkson–Kruskal symmetry reduction of Eq. (4). Following Bateman (1943), a solution to Eq. (9) can be constructed by assuming $V_{j}(z(t))=\exp \left( ip z(t)\right) v_{j}(p)$, $i=\sqrt{-1}$, $p\in {\mathbb {R}}$, thereby yielding the reduction

10 $$\begin{aligned} {\left\{ \begin{array}{ll} V_{j}(z(t))=\exp \left( ip z(t)\right) v_{j}(p) ;\\ 0=ip v_{j}+v_{j+1}\pm v_{j+2} , \quad {\mathrm{if}} \quad \xi (t)=\mp bt+\varpi ,\\ \end{array}\right. } \end{aligned}$$

which has the solution

11 $$\begin{aligned} {\left\{ \begin{array}{ll} V_{j}(z(t))=\exp \left( ip z(t)\right) v_{j}(p) ;\\ \, v_{j}=2^{-j}\left( \left( \mp 1-\sqrt{1\mp 4ip} \right) ^{j}C_{1}+\left( \mp 1+\sqrt{1\mp 4ip} \right) ^{j}C_{2}\right) ,\\ \, \xi (t)=\mp bt+\varpi ,\\ \end{array}\right. } \end{aligned}$$

$C_{1}$ and $C_{2}$ being arbitrary constants. In summary, we have the following result.

#### **Theorem 1**

*Differential-difference equation *(4)* has solutions*

12 $$A_{j}(t) = \left\{ {\begin{array}{ll} \Upsilon _{0}\exp ( 2bt)+2^{-j}\Big [\left( -\omega _{-} \right) ^{j}C_{1}+\left( \omega _{-}-2\right) ^{j}C_{2}\Big ]\exp (-ipbt ) &\quad \xi (t)=-bt+\varpi \\ \Upsilon _{0}+2^{-j}\Big [\left( 2-\omega _{+}\right) ^{j}C_{1}+\omega _{+ }^{j}C_{2}\Big ]\exp (ipbt) &\quad \xi (t)=bt+\varpi \\ \end{array} } \right.$$

*where*$$\omega _{\pm }=1+\sqrt{1\pm 4ip}$$*, and p,*$$\varpi ,$$$$\Upsilon _{0},$$$$C_{1},$$$$C_{2}$$* are constants.*

For $\xi (t)=-bt+\varpi$, it is observed that condition  (3a) affords the vector of parameters and arbitrary constants $\zeta :=\left[ p,\Upsilon _{0},C_{1},C_{2}\right]$ values $[-2i,-(C_{1}+C_{2}),C_{1},C_{2}]$, $[p,0,-C_{2},C_{2}]$ or $[0,0,C_{1},C_{2}]$. In the instance $\xi (t)=bt+\varpi$, however, condition (3a) admits only the vector $[0,-(C_{1}+C_{2}),C_{1},C_{2}]$. Enforcing the remaining conditions  (3b) and (3c) yields the following result.

#### **Corollary 1**

*The differential-difference equation* (4)* and conditions*  (3a)* to* (3c)* are verified by*

13 $$\begin{aligned} A_{j}(t)= {\left\{ \begin{array}{ll} T^{\star } &\quad j=0\\ {\displaystyle (\mp 1)^{j} } &\quad j\ge 1 ,\\ \end{array}\right. } \end{aligned}$$

*for*$$\xi (t)=\mp bt+\varpi,$$$$\Psi (t)$$* being of the form*$${\displaystyle \Psi (t)=T^{\star }-1+\exp (bt+\varpi )}.$$

### A variant Clarkson–Kruskal similarity reduction

Equation (6) can be alternatively pictured as the system of uncoupled equations

14 $$\begin{aligned} {\left\{ \begin{array}{ll} 0=\Upsilon ^{'}(t)+ \left[ \xi ^{ '}(t)-b\right] \Upsilon (t) ,\\ {\displaystyle 0=\Theta (t)z^{'}(t)\frac{dV_{j}(z)}{dz(t)}+\Theta ^{'}(t)V_{j}(z(t))+\xi ^{ '}(t)\Theta (t)V_{j+1}(z(t))-b\Theta (t)V_{j+2}(z(t)) ,}\\ \end{array}\right. } \end{aligned}$$

which can be recast as

15a $$\begin{aligned} \Upsilon (t)&=\Upsilon _{0}\exp \Big (bt-\xi (t)\Big ) ; \end{aligned}$$

15b $$\begin{aligned}&{\displaystyle 0=\Theta ^{'}(t)\Theta ^{-1}(t)V_{j}(z(t))+\xi ^{'}(t)V_{j+1}(z(t)) -bV_{j+2}(z(t))+z^{'}(t)\frac{dV_{j}(z)}{dz}}. \end{aligned}$$

Here we shall only study the special case of Eqs. (14) and  (15) for which $z(t)=t$; rather than Eq. (15b), the following equation is studied

16 $$\begin{aligned} 0=\Theta ^{'}(t)\Theta ^{-1}(t)V_{j}(t)+ \xi ^{'}(t)V_{j+1}(t)-bV_{j+2}(t)+\frac{dV_{j}(t)}{dt}. \end{aligned}$$

Next, we shall furnish a difference equation reformulation of Eq. (16) by employing the variant Clarkson–Kruskal ansatz

17 $$\begin{aligned} V_{j}(t)=g_{j}(t) v_{j}, \end{aligned}$$

$g_{j}$ being a continuously differentiable function of *t* for each *j*. Substituting ansatz (17) into differential-difference equation Eq. (15b) yields

18 $$\begin{aligned}&0= \left( 1+\gimel _{j}(t)\right) v_{j}+\beth _{j}(t)v_{j+1}-b \daleth _{j}(t)v_{j+2} ;\nonumber \\&\gimel _{j}(t):=g_{j}(t) g_{j}^{ '-1}(t)\frac{\Theta ^{'}(t)}{\Theta (t)} , \quad\beth _{j}(t):=g_{j+1}(t) g_{j}^{ '-1}(t) \xi ^{'}(t),\nonumber \\&\quad \daleth _{j}(t):=g_{j+2}(t) g_{j}^{ '-1}(t). \end{aligned}$$

It is clear that our present objective can be attained by imposing conditions on Eq. (18) which simultaneously make $\gimel _{j}(t)$, $\beth _{j}(t)$ and $\daleth _{j}(t)$ constant in *t*. One such way is to, firstly, endow $g_{j}(t)$ with a recurrence

19 $$\begin{aligned} g_{j}(t)=\xi ^{' j}(t)g_{0}(t) , \end{aligned}$$

from which it follows that $\beth _{j}(t)$ and $\daleth _{j}(t)$ are equivalent-

20 $$\begin{aligned} \beth _{j}(t)=\daleth _{j}(t)=\Big (\xi ^{'-2}(t) g_{0}^{-1}(t)g_{0}^{'}(t)+j\xi ^{-3}(t)\xi ^{''}(t)\Big )^{-1} ; \end{aligned}$$

and

21 $$\begin{aligned} \gimel _{j}(t)=\daleth _{j}(t) \xi ^{'-2}(t) \Theta ^{'}(t)\Theta ^{-1}(t), \end{aligned}$$

the derivative of $g_{j}(t)$ with respect to *t* being

22 $$\begin{aligned} g_{j}^{'}(t)=\xi ^{'j}(t)g_{0}^{'}(t)+j\xi ^{' j-1}(t)\xi ^{''}(t) g_{0}(t). \end{aligned}$$

Incorporating Eqs. (19)–(22) into Eq. (18) transforms it into

23 $$\begin{aligned} {\left\{ \begin{array}{ll} {\displaystyle 0= \left( 1+\daleth _{j}(t) \xi ^{'-2}(t) \Theta ^{'}(t)\Theta ^{-1}(t)\right) v_{j}+\daleth _{j}(t)v_{j+1}-b \daleth _{j}(t)v_{j+2} ;}\\ {\displaystyle \daleth _{j}(t):=\Big (\xi ^{'-2}(t)g_{0}^{-1}(t)g_{0}^{'}(t)+j\xi ^{-3}(t) \xi ^{''}(t)\Big )^{-1} .}\\ \end{array}\right. } \end{aligned}$$

Secondly, requiring that $\Theta ^{'}(t)\Theta ^{-1}(t)$ be of a constant proportion to $\xi ^{' 2}(t)$, that is

24 $$\begin{aligned} \Theta ^{'}(t)\Theta ^{-1}(t)=q \xi ^{' 2}(t) ,\quad q\ne 0 , \end{aligned}$$

converts Eq. (23) into

25 $$\begin{aligned} {\left\{ \begin{array}{ll} 0= \left( 1+q\daleth _{j}(t) \right) v_{j}+\daleth _{j}(t)v_{j+1}-b \daleth _{j}(t)v_{j+2} ;\\ {\displaystyle \daleth _{j}(t):=\Big (\xi ^{'-2}(t)g_{0}^{-1}(t)g_{0}^{'}(t)+j\xi ^{-3}(t)\xi ^{''}(t)\Big )^{-1} .}\\ \end{array}\right. } \end{aligned}$$

Finally, we shall enforce a set of constraints which makes $\daleth _{j}(z(t),t)$ constant with respect to the variable *t*: We set

26 $$\begin{aligned} \xi ^{''}(t)\xi ^{'-3}(t)=p,\quad \xi ^{'-2}(t)g_{0}^{-1}(t)g_{0}^{'}(t)=1 . \end{aligned}$$

This yields the following reduction of Eq. (15b):

27 $$\begin{aligned} {\left\{ \begin{array}{ll} {\displaystyle V_{j}(t)=\xi ^{' j}(t)g_{0}(t) v_{j} ;}\\ {\displaystyle g_{0}(t)=C_{9}\exp \left( \int _{t} \xi ^{' 2}(\tau )d\tau \right) ; \Theta (t)=C_{10}\exp \left( q\int _{t} \xi ^{'2}(\tau ) d\tau \right) ;}\\ {\displaystyle \xi (t)=r+\frac{1-s\sqrt{s^{-2}-2pt}}{ps} , \xi (0)=r , \lim _{t\rightarrow 0} \xi ^{'}(t)=s ,}\\ {\displaystyle r,s\in {\mathbb {R}}\cup \{+\infty \} ;}\\ {\displaystyle 0=(pj+(q+1)){\tilde{v}}_{j}+(j+1){\tilde{v}}_{j+1}-b(j+1)(j+2){\tilde{v}}_{j+2} ,}\\ \, {\tilde{v}}_{j}=v_{j}/j! ;\\ \end{array}\right. } \end{aligned}$$

where $C_{9}$ and $C_{10}$ are integration constants. It is worth observing that Eqs. (5), (15a) and (27) constitute another Clarkson–Kruskal similarity reduction of ${\mathcal {D}}\Delta {\mathcal {E}}$ (1).

The first few terms of the solution sequence $\left\{ {\tilde{v}}_{j}\right\} _{0}^{\infty }$ satisfying the difference equation of Eq. (27) are

28 $$\begin{aligned} {\tilde{v}}_{0}=\kappa _{1} ; {\tilde{v}}_{1}=\kappa _{2} ;&{\tilde{v}}_{2}=\frac{\kappa _{1}(1+q)+\kappa _{2}}{2b} ;\nonumber \\&{\tilde{v}}_{3}=\frac{(1+q)\kappa _{1}+(1+b(1+p+q))\kappa _{2}}{6b^{2}} ; \ldots , \end{aligned}$$

where $\kappa _{1}$ and $\kappa _{2}$ are arbitrary constants. A closed solution to the difference equation can be sought in the sense of Popenda (1987) or Mallik (1997). However the solutions to this class of variable coefficient difference equations are known to be quite cumbersome, and as such for practical purposes the generators of $\left\{ {\tilde{v}}_{j}\right\} _{0}^{\infty }$ are constructed instead.

Suppose that ${{\mathsf Z}}(x)$ is the sought generating function,

29 $$\begin{aligned} {{\mathsf Z}}(x)=\sum _{j=0}^{\infty } {\tilde{v}}_{j} x^{j} , \end{aligned}$$

of the sequence $\left\{ {\tilde{v}}_{j}\right\} _{0}^{\infty }$. Then,

30 $$\begin{aligned} {{\mathsf Z}}^{'}(x)&=\sum _{j=0}^{\infty } (j+1) {\tilde{v}}_{j+1} x^{j} ;\nonumber \\ {{\mathsf Z}}^{''}(x)&=\sum _{j=0}^{\infty } (j+1)(j+2) {\tilde{v}}_{j+2} x^{j}; {and}\nonumber \\ \omega x {{\mathsf Z}}^{'}(x)&=\sum _{j=0}^{\infty } \omega j {\tilde{v}}_{j} x^{j},\quad \omega \in {\mathbb {R}} . \end{aligned}$$

Multiplying through the difference equation in Eq. (27) by $x^{j}$, summing from *j* value 0 to $\infty$ and applying Eqs.  (29) and (30), it is realized that the generating ordinary differential equation for the solution sequence $\left\{ {\tilde{v}}_{j}\right\} _{0}^{\infty }$ of the difference equation in Eq. (27) is

31 $$\begin{aligned} {\left\{ \begin{array}{ll} 0=(1+q) {{\mathsf Z}}(x)+(1+p x) {{\mathsf Z}}^{ '}(x)-b {{\mathsf Z}}^{ ''}(x) ;\\ {{\mathsf Z}}(0)=C_{1} , {{\mathsf Z}}^{ '}(0)=C_{2} .\\ \end{array}\right. } \end{aligned}$$

The solution to Eq. (31), which is the generator of the sequence $\left\{ {\tilde{v}}_{j}\right\} _{j=0}^{\infty }$ , is given by

32 $$\begin{aligned}&{\displaystyle {{\mathsf Z}}(x)}=\nonumber \\&\quad {\left\{ \begin{array}{ll} {\displaystyle C_{1}+C_{2}\sqrt{\frac{2b}{p}}\left[ -{{\mathcal {D}}}\left( \frac{1}{\sqrt{2bp}}\right) +\exp \left( \frac{x(2+px)}{2b}\right) {{\mathcal {D}}}\left( \frac{1+px}{\sqrt{2bp}}\right) \right] } &\quad q=-1 ;\\ {\displaystyle C_{3} {{\mathcal {H}}}_{-\frac{1+q}{p}}\left( \frac{1+p x}{\sqrt{2bp}}\right) +{C_{4}} \,\,\,{}_{1}{{\mathcal {F}}}_{1}\left( \frac{1+q}{2p},\frac{1}{2},\frac{(1+p x)^{2}}{2bp}\right) } &\quad q\ne -1 ,\\ \end{array}\right. } \end{aligned}$$

where ${{\mathcal {D}}}$ is the Dawson function defined by ${\displaystyle {{\mathcal {D}}}(x)=\exp (-x^{2})\int _{0}^{x} \exp (y^{2})dy}$ or through the error function ${\text {Erf}}$ as ${\displaystyle {{\mathcal {D}}}(x)=\frac{\sqrt{\pi }}{2}\exp (-x^{2}) {\text {Erf}}(x)}$; ${{\mathcal {H}}}_{r}(x)$ is *r*th Hermite *polynomial* which satisfies the ordinary differential equation

33 $$\begin{aligned} \frac{d^{2}y(x)}{dx^{2}}-2x\frac{dy(x)}{dx}+2ry(x)=0 , \quad r\in {{\mathbb {R}}} ; \end{aligned}$$

$_{1}{{\mathcal {F}}}_{1}$ is the Kummer confluent hypergeometric function defined by

34 $$\begin{aligned} {}_{1}{{\mathcal {F}}}_{1}\left( a,b,x\right) =\sum _{j=0}^{\infty } \frac{\left( a\right) _{j}}{\left( b\right) _{j}}\frac{x^{j}}{j!} ,\quad a,b\in {{\mathbb {R}}}^{+}\cup \left\{ 0\right\} ; \end{aligned}$$

with ${\displaystyle C_{3}=\frac{{{\mathsf U}}(p,q)}{{{\mathsf W}}(p,q)}}$ and ${\displaystyle C_{4}=\frac{{{\mathsf V}}(p,q)}{{{\mathsf W}}(p,q)}}$. In the aforegone, ${{\mathsf U}}(p,q)$, ${{\mathsf V}}(p,q)$ and ${{\mathsf W}}(p,q)$ are, respectively,

35 $$\begin{aligned} {\left\{ \begin{array}{ll} {{\mathsf U}}(p,q)={\displaystyle -b p  \,\,\,{}_{1}{{\mathcal {F}}}_{1}\left( \frac{1+q}{2 p},\frac{1}{2},\frac{1}{2 b p}\right) C_{2} +(1+q)\, _{1}{{\mathcal {F}}}_{1}\left( \frac{1+2 p+q}{2 p},\frac{3}{2},\frac{1}{2 b p}\right) C_{1} ,}\\ {{\mathsf V}}(p,q)={\displaystyle b p {{\mathcal {H}}}_{-\frac{1+q}{p}}\left( \frac{1}{\sqrt{2bp}}\right) C_{2}+(1+q)\sqrt{2bp} {{\mathcal {H}}}_{-\frac{1+p+q}{p}}\left( \frac{1}{\sqrt{2bp} }\right) C_{1} ,}\\ {{\mathsf W}}(p,q)={\displaystyle (1+q) \Bigg [\sqrt{2} \sqrt{b} \sqrt{p} {{\mathcal {H}}}_{-\frac{1+p+q}{p}}\left( \frac{1}{\sqrt{2bp}}\right)\, _{1}{{\mathcal {F}}}_{1}\left( \frac{1+q}{2 p},\frac{1}{2},\frac{1}{2 b p}\right) +}\\ { \displaystyle {{\mathcal {H}}}_{-\frac{1+q}{p}}\left( \frac{1}{\sqrt{2bp}}\right)\, _{1}{{\mathcal {F}}}_{1}\left( \frac{1+2 p+q}{2 p},\frac{3}{2},\frac{1}{2 b p}\right) \Bigg ] ,}\\ \end{array}\right. } \end{aligned}$$

$C_{1}$ and $C_{2}$ being arbitrary constants.

The above results can be summarized as the following.

#### **Theorem 2**

*The differential-difference equation* (4)* has a solution*

36 $$\begin{aligned} {\left\{ \begin{array}{ll} {\displaystyle A_{j}(t)=\Upsilon _{0}\exp \Big (bt-\xi (t)\Big )+\Lambda \exp \left( (q+1)\int _{t} \xi ^{'2}(\tau ) d\tau \right) \xi ^{'j}(t)j! {\tilde{v}}_{j} ;}\\ {\displaystyle \xi (t)=r+\frac{1- s\sqrt{s^{-2}-2pt}}{ps} ,\quad \xi (0)=r , \lim _{t\rightarrow 0} \xi ^{'}(t)=s ;}\\ \end{array}\right. } \end{aligned}$$

*where m, p, q, r, s,*$$\Upsilon _{0},$$$$\Lambda$$*are constants, and*$${\tilde{v}}_{j}$$*is generated by the function in Eq.* (32)*.*

If the constraints due to Eqs. (3a) to (3c) are further imposed on solutions given by Theorem 2, $A_{j}$s which are potentially applicable in solving Eq. (1) are obtained. Condition (3a) implies that $\Upsilon _{0}$, $q=-1$ and $\Lambda =T^{\star }/{\tilde{v}}_{0}$. Further, Eq.  (3c) shows that ${\tilde{v}}_{1}$ is expressible in terms of ${\tilde{v}}_{0}$ as ${\tilde{v}}_{1}=\left( \gamma \kappa {\tilde{v}}_{0}\right) /\left( \lambda T^{\star }\right)$. Consequent upon these observations, we have the following special case of Theorem 2.

#### **Corollary 2**

*The differential-difference equation* (4)* and conditions*  (3a)*to* (3c)* are satisfied by*

37 $$\begin{aligned} A_{j}(t)=T^{\star }j! \xi ^{'j}(t)\frac{{\tilde{v}}_{j}}{{\tilde{v}}_{0}} , \end{aligned}$$

$\Psi (t)$* being of the form*${\displaystyle \Psi (t)=T^{\star }\sum\nolimits_{j=0}^{\infty }\Bigg (\left( r-\frac{1}{p}\right) +\frac{1}{p\sqrt{1-2ps^{2}t}}\Bigg )^{j}\frac{{\tilde{v}}_{j}}{{\tilde{v}}_{0}} ,}$* and*$\left\{ {\tilde{v}}_{j}\right\} _{0}^{\infty }$*is generated by Eq.* (32)*,*$q=-1.$

## Application to the plate solidification problem

### Traveling wave solutions

In this subsection, Theorem 1 is applied in solving Eq. (1). This approach is only admissible provided the range of convergence

38 $$\begin{aligned} -\lim _{j\rightarrow \infty }\left| \frac{(j+1)A_{j}(t)}{A_{j+1}(t)}\right| +\Phi (t)<x<\lim _{j\rightarrow \infty } \left| \frac{(j+1)A_{j}(t)}{A_{j+1}(t)}\right| +\Phi (t) \end{aligned}$$

of series (2), per time, contains the spatial domain $\Phi (t)< x< {\bar{x}}$ of Eq. (1). Elementary calculations confirm this in the affirmative: In point of fact, the range of convergence of the series is the set of real numbers. Due to the fact that $\Phi (t)={\bar{x}}-\xi (t)$, $x={\bar{x}}$ being the plate’s surface, we shall only consider $\xi (t)$ in the form

39 $$\begin{aligned} \xi (t)=bt+\varpi . \end{aligned}$$

Further, the nature of solution (12)$_{2}$ suggests the existence of a traveling wave solution to the Stefan problem  (1), one which is derived by substituting it into Eq. (2):

40 $$\begin{aligned} {\left\{ \begin{array}{ll} T(x,t)=\Upsilon _{0}\exp \left( x-\Phi (t)\right) \\ +\exp \Bigg (ipbt-\frac{1}{2}\left( -1+\sqrt{1+4ip} \right) (x-\Phi (t))\Bigg )\\ \times \Bigg [C_{1}+C_{2}\exp \left( \left( \sqrt{1+4ip} \right) (x-\Phi (t))\right) \Bigg ] ;\\ \xi (t)=+bt ; \Phi (t)={\bar{x}}-b t ,\\ \end{array}\right. } \end{aligned}$$

up to integration constants $\Upsilon _{0}$, $C_{1}$ and $C_{2}$.

Solving Stefan problem (1) up to initial and boundary conditions demands the application of Corollary  1. This leads to the special case of plate solidification process Eq. (1) for which the temperature of the surface of the plate differs from that of the constant temperature $T^{\star }$ of the solidification front by a magnitude ${\displaystyle \left (-1+\exp \left( b t\right) \right )}$ :

41a $$\partial _{t}T=b \partial _{xx}T \quad {\text{in}}\, \left( 0,\Phi (t)\right) \times \left[ 0,\tau \right];$$

41b $$\begin{aligned} \,\Phi (t)={\bar{x}}-\xi (t) ,\quad \xi (0)=0 ; \end{aligned}$$

41c $$\begin{aligned} T\Big |_{x={\bar{x}}}=T^{\star }-1+\exp \left( b t\right) \quad {\mathrm{for}}\,t\in \left[ 0,\tau \right] ; \end{aligned}$$

41d $$\begin{aligned} T\Big |_{x=\Phi (t)}=T^{\star } ; -\lambda \partial _{x}T\Big |_{x=\Phi (t)}=\gamma \kappa d_{t}\Phi (t) \quad {\mathrm{for}}\,t\in \left[ 0,\tau \right] . \end{aligned}$$

Upon a reflection of the analyses of the previous Section and the preceding paragraph of this Section, it is observed that Eq. (41) has the exact closed travelling wave solution

42 $$\begin{aligned} {\left\{ \begin{array}{ll} {\displaystyle T(x,t)=T^{\star }-1+\exp \Big (x-\Phi (t)\Big ) ;}\\ \Phi (t)={\bar{x}}-\xi (t) ;\quad \xi (t)=+bt ,\\ \end{array}\right. } \end{aligned}$$

with speed *b*, the value of the thermal diffusivity.

### Similarity solutions

The study of similarity solutions to the solidification problem due the analyses of “Clarkson–Kruskal’s similarity reduction and explicit solutions” section is hinged on the nature of the second solution of the ${\mathcal {D}}\Delta {\mathcal {E}}$ (4) as given by Theorem 2. Consequent upon Eq. (36), similarity solutions of the form

43 $$\begin{aligned} T(x,t)=\exp \Bigg ((q+1)\int \xi ^{' 2}(t) dt\Bigg ) \sum _{j=0}^{\infty } {\tilde{v}}_{j}\Bigg (1+\frac{(x-{\bar{x}})}{\xi ^{'-1}(t)}\Bigg )^{j} , \end{aligned}$$

which are self-similar in variables ${\displaystyle T(x,t)/\exp \left( (q+1)\int \xi ^{' 2}(t) dt\right) }$ and ${\displaystyle x/\xi ^{'-1}(t)}$, are obtained.

In this instance the thickness of the solidified layer $\xi (t)$ is expected to satisfy $\xi (0)=0$, the behaviour of its gradient being unspecified at $t=0$. Suppose that $\xi ^{'}(0)=s\in \left\{ -\infty ,+\infty \right\}$. Since

44 $$\begin{aligned} \xi (t)=u-\frac{1}{p}\sqrt{p^{2}u^{2}-2p t} ;\quad u=\frac{1}{ps} , \end{aligned}$$

the solidification front evolution $\Phi (t)$ then takes the form

45 $$\begin{aligned} \Phi (t)={\bar{x}}-\sqrt{\frac{-2 t}{p}} ;\quad p<0 . \end{aligned}$$

The choice of parameters *p* and *q* such that the flux condition Eq. (1e) is satisfied is contingent on ${\mathcal {D}}\Delta {\mathcal {E}}$ solution (36). From Eq. (2), it follows that $\partial _{x}T\Big |_{x=\Phi (t)}=A_{1}(t)$, and it is observed that if the integration constant $\Upsilon _{0}$ of (36) is set to zero,

46 $$\begin{aligned} {\displaystyle A_{1}(t)\propto t^{\mu } ,\quad \mu =-\frac{1+p+q}{2p} .} \end{aligned}$$

However the right hand side of Eq. (1e) is proportional to $\root -2 \of {t}$, thereby implying that $q=-1$ (for consistency) and yielding the similarity solution to Eq. (1) as

47 $$\begin{aligned} {\left\{ \begin{array}{ll} {\displaystyle T(x,t)=\sqrt{t}\Bigg [\sqrt{-\frac{1}{b t}} x C_{1}+\frac{x}{2}\sqrt{\frac{\pi }{b}} \exp \left( -\frac{x^{2}}{4b t}\right) \quad {\mathrm{Erf}}\quad \left( \frac{x}{2\sqrt{b t}}\right) C_{2}\Bigg ] ,}\\ {\displaystyle \xi (t)=\sqrt{\frac{-2 t}{p}} ; \Phi (t)={\bar{x}}-\sqrt{\frac{-2 t}{p}} ; \quad p<0 ,}\\ \end{array}\right. } \end{aligned}$$

up to arbitrary constants *p*, $C_{1}$ and $C_{2}$ . An alternative approach, one which consists of the application of Corollary  2, could be employed to reach the same result as given by Eq. (47).

The mode of determination of the arbitrary constants in Eq. (47) relies on the nature of admissible temperature $\Psi (t)$ of the plate’s surface. If the surface of the plate is kept at a constant temperature $\Psi _{c}$ , say, then the solidification problem (1) has the solution

48 $$\begin{aligned} {\left\{ \begin{array}{ll} {\displaystyle T(x,t)=\Psi _{c}-i \sqrt{\frac{ \pi }{2bp}} \exp \left( -\frac{1}{2bp}\right) \quad {\mathrm{Erf}}\left( \frac{x-{\bar{x}}}{2\sqrt{b t}}\right) ,}\\ {\displaystyle \xi (t)=\sqrt{\frac{-2 t}{p}} ; \quad p<0 ,}\\ \end{array}\right. } \end{aligned}$$

where ${\mathrm{Erf}}(\cdot )$ is the error function and *p* is a negative root of the transcendental equation

49 $$\begin{aligned} {\displaystyle \Psi _{c}-T^{\star }=\frac{\gamma \kappa }{\lambda p}\,\,\,{}_{1}{{\mathcal {F}}}_{1}\Bigg (1 , \frac{3}{2} ; -\frac{1}{2bp}\Bigg ) ,\quad p<0 .} \end{aligned}$$

On the other hand, if the temperature of the surface of the plate varies in time, then the solidification problem (1) has the solution

50 $$\begin{aligned} {\left\{ \begin{array}{ll} {\displaystyle T(x,t)=T^{\star }+\sqrt{\frac{\pi }{2bp}} \Bigg [i \, {\mathrm{Erf}}\, \left( \frac{x-{\bar{x}}}{2\sqrt{b t}}\right) +{\mathrm{Erfi}}\left( \frac{1}{\sqrt{2bp}}\right) \Bigg ] ,}\\ {\displaystyle \xi (t)=\sqrt{\frac{-2 t}{p}} ;\quad p<0 ,}\\ \end{array}\right. } \end{aligned}$$

where ${\mathrm{Erfi}}(\cdot )$ is the imaginary error function defined by ${\mathrm{Erfi}}(x)=-i{\mathrm{}}{\mathrm{Erf}}(ix)$ and $p=p(;t)$ which is the negative root, per time, of the equation

51 $$\begin{aligned} {\displaystyle \Psi (t)-T^{\star }=\frac{\gamma \kappa }{\lambda p(;t)}\,\,\,{}_{1}{{\mathcal {F}}}_{1}\Bigg (1 , \frac{3}{2} ; -\frac{1}{2bp(;t)}\Bigg ) ,\quad p<0 .} \end{aligned}$$

The notation *p*(; *t*) in Eq. (51) denotes that *p* is determined per time throughout the solidification process unlike the case described by Eq. (49). It is also worth noting that the temperature distributions given by Eqs. (48) and  (50) are real-valued due to the strict negativity of *p*.

### Illustration

In this Subsection, we shall illustrate some of the results obtained in the earlier parts of the Section. Suppose that the material under consideration is the same as chosen by Grzymkowski et al. (2013), having thermal conductivity 25 W/mK, latent heat of fusion 247,000 J/kg, density 7000 kg/m$^{3}$, with ${\bar{x}}=0.2000$ and $T^{\star }=1773.15$ K. The first example concerns a traveling wave solution of Problem (1); the second example deals with Problem (1) when the moving front has a parabolic shape of the form (45), with constant temperature $\Psi$ at the fixed end ${\bar{x}}$ and solution (48); while the third is similar to the second, but with a time-varying fixed end temperature $\Psi (t)$.

#### *Example 1*

Suppose that $\varpi =0$ and the fixed edge $x={\bar{x}}$ is kept at the slightly varying temperature $1772.1500 +\exp \Big ({t/69,160,000}\Big )$, then the temperature distribution of the solid phase of the solidifying material is given by

52 $$\begin{aligned} {\left\{ \begin{array}{ll} {\displaystyle T(x,t)=1772.1500 +\exp \Big ({-0.2000+\frac{t}{69,160,000}+x}\Big )} ,\\ {\displaystyle \Phi (t)=0.2000 -\frac{t}{69,160,000} ,}\\ \end{array}\right. } \end{aligned}$$

the time for complete solidification, which is obtained by setting $\Phi (t)$ to zero, being $1.3832\times 10^7$ s.

#### *Example 2*

Let $\varpi =0$ and the fixed edge ${\bar{x}}=0.2000$ of the solidifying material is kept at the constant temperature 1876.9200*K*. From Eq. (49), *p* can be evaluated using the ${\mathtt {FindRoot}}$ command of the symbolic computation software Mathematica to be $-10^{7}$. The temperature distribution of the solid phase is given by

53 $$\begin{aligned} {\left\{ \begin{array}{ll} {\displaystyle T(x,t)=1876.9200 -104.6590\, {\text {Erf}}\,\left( \frac{4158.1200 \,(-0.2000+x)}{\sqrt{t}}\right) } ,\\ {\displaystyle \Phi (t)=0.2000 -0.000447214 \sqrt{t} ,}\\ \end{array}\right. } \end{aligned}$$

total solidification in this case occurring in $2.0000\times 10^5$ s.

#### *Example 3*

Consider the instance in which the fixed edge ${\bar{x}}=0.2000$ is subjected to a periodic temperature $\Psi (t)=1876.92\sin (t)$ units. Here, *p*(; *t*) values are computed, per time, by employing Eq.  (51). In the current example, *p*(; *t*) values at selected times varying from time period 3600–21,600 s are given by Table 1. It is observed that despite the periodic nature of the temperature at the fixed edge both *p*(; *t*) and $\Phi$ are, respectively, in time, monotonic increasing and decreasing functions. The temperature at each point in time can be evaluated through Eq. (50).

**Table 1:** Values of *p* and $\Phi (t)$ at selected periods during solidification in Example 3

*t* (s)	3600	7200	10,800	14,400	18,000	21,600
$p \,(\times 10^{ 9})$	−3.0991	−2.5858	−2.2667	−2.0729	−1.9697	−1.9404
$\Phi\,(t)$ (*m*)	0.1984	0.1976	0.1969	0.1962	0.1957	0.1952

## Conclusion

In the present paper similarity reductions of a ${\mathcal {D}}\Delta {\mathcal {E}}$, with a variable coefficient which is a function of the continuous variable, arising from a solidification process were derived using discrete Clarkson–Kruskal techniques. These reductions admit two classes of the variable coefficient (which determines the nature of the solidification front), thereby leading to distinct solutions of the ${\mathcal {D}}\Delta {\mathcal {E}}$. An application of these ${\mathcal {D}}\Delta {\mathcal {E}}$ solutions to the solidification problem revealed the presence of planar or parabolic interfacial front, respectively, when the temperature distribution in the solid phase is of the traveling wave or similarity type.

The application of the results obtained can be extended to solidification and melting processes in other simple geometries, directly so the spherical. While the results obtained herein are by no means exhaustive, as there remains interesting lines of study concerning exact analyses and qualitative properties of Eq. (1) for arbitrary continuously differentiable $\xi (t)$ yet unconsidered, the approach presented herein offers fresh and insightful perspectives for exact analyses of differential-difference equations as well as solidification problems.
